# Intercomparison of the LBIR Absolute Cryogenic Radiometers to the NIST Optical Power Measurement Standard

**DOI:** 10.6028/jres.111.024

**Published:** 2006-08-01

**Authors:** James A. Fedchak, Adriaan C. Carter, Raju Datla

**Affiliations:** Jung Research and Development Corp., 1706 U St., Washington DC 20009, USA; National Institute of Standards and Technology, Gaithersburg, MD 20899-8441

**Keywords:** absolute cryogenic radiometer, ACR, calibration, electrical substitution radiometers, HACR, high accuracy cryogenic radiometer, intercomparison, LBIR, optical power measurement standard, POWR, primary optical watt radiometer, traceability

## Abstract

The Low Background Infrared calibration (LBIR) facility at the National Institute of Standards and Technology (NIST) presently maintains four absolute cryogenic radiometers (ACRs) which serve as standard reference detectors for infrared calibrations performed by the facility. The primary standard for optical power measurements at NIST-Gaithersburg has been the High Accuracy Cryogenic Radiometer (HACR). Recently, an improved radiometer, the Primary Optical Watt Radiometer (POWR), has replaced the HACR as the primary standard. In this paper, we present the results of comparisons between the radiometric powers measured by the four ACRs presently maintained by the LBIR facility to that measured by the HACR and POWR. This was done by using a Si photodiode light-trapping detector as a secondary transfer standard to compare the primary national standards to the ACRs maintained by the LBIR facility. The technique used to compare an ACR to the trap detector is described in detail. The absolute optical power measurements are found to be within 0.1 % of the primary standard for all the ACRs examined in this study.

## 1. Introduction

The Low Background Infrared calibration (LBIR) facility at NIST is responsible for the infrared power measurement standard in background environments with temperatures lower than 80 K. Broadband radiometric measurements that cover the entire infrared spectrum are used primarily for the calibration of blackbody sources. Spectral calibrations of detectors can also be performed over infrared wavelengths ranging from 2 µm to 30 µm. The standard reference detectors used in the LBIR facility are electrical substitution radiometers operated near 2 K, known as the absolute cryogenic radiometers (ACRs). Two generations of ACRs are presently in use in the LBIR facility: ACR I and ACR II. The ACR II was designed to have higher sensitivity, higher absorptance, and faster time response than the ACR I [1]. Presently the LBIR facility maintains two ACR I and two ACR II radiometers. The ACR I radiometers have larger receiver cones and can measure higher optical powers than the ACR II radiometers; the ACR I radiometers are maintained for use in situations where these characteristics are required. All absolute radiometric power measurements performed by the LBIR facility are ultimately based on a cross calibration between the ACRs and the optical power standard at NIST-Gaithersburg. For the infrared calibrations performed at the LBIR facility, a calibration uncertainty of less than 0.1 % is required for the ACRs.

It is important to periodically check the ACRs maintained by the LBIR facility against the primary standard. The primary standard for optical power was the High Accuracy Cryogenic Radiometer (HACR) [2]. During this intercomparison effort the older NIST primary standard, the HACR, was replaced by the newer Primary Optical Watt Radiometer (POWR) [3]. Both of these instruments are electrical substitution radiometers operated at liquid helium temperatures. It is not possible or, at least, not practically feasible, to directly compare the ACRs to the primary standard. Instead, a Si photodiode light-trapping detector was used as a transfer standard to compare the accuracy of the ACRs to each other and to the primary standard. The trap detector in this study was paired with a particular transimpedance amplifier. The external responsivity of the trap detector and amplifier pair (from here forward simply called the trap detector) was determined at λ=632.8 nm by comparing the power of a He-Ne laser, as measured by the primary national standard, to the voltage output of the transimpedance amplifier set at a fixed gain. The comparison of the trap detector to the LBIR facility ACRs was then performed in a similar way: The optical power of a He-Ne laser beam was determined by the voltage output from the trap detector. This was then compared to the radiometric power measured by one of the ACRs maintained by the LBIR facility. The process of comparing the absolute radiometric power measured by an ACR to that determined from the responsivity of a photodiode trap detector is referred to as an intercomparison. Over the course of the intercomparison effort, the primary optical power measurement standard was transferred from HACR to POWR. The particular trap used in this intercomparison effort was initially calibrated by HACR when the effort started, and was calibrated again by POWR at the end. The calibration of the trap as characterized by the two different primary optical power measurement standards agreed to 0.002 % ± 0.028 % (*k* =1).

Comparisons between ACRs maintained by the LBIR facility and trap detectors have been performed in the past [4,5]. Until now, there have not been published results from an intercomparison of an ACR to a trap detector directly calibrated by the primary standard. Previous comparisons utilized an uncalibrated trap detector that was either assumed to have a quantum efficiency of unity [4], or was subsequently calibrated against a second transfer radiometer which was calibrated against the HACR [5]. In this work, all of the ACRs presently in the LBIR facility are intercompared to the HACR and POWR. Since the previous intercomparisons at the LBIR [4,5] facility, several improvements in the optical setup used by the LBIR facility to compare the ACRs to the trap detector, as well as improvements in the primary standard facility, have allowed smaller uncertainties in the intercomparison than in the past.

## 2. Apparatus

### 2.1 ACR

**Fig. 1:**
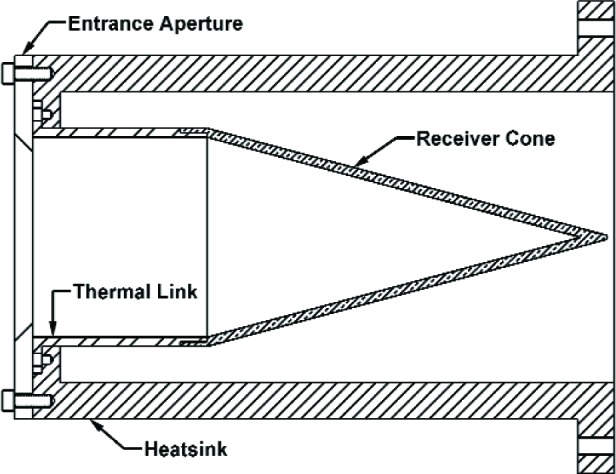
Cross section view of an ACR.

Two different generations of ACRs were investigated in this study: ACR I and ACR II. ACR II was developed to have an improved sensitivity over ACR I, but the overall design is similar. The design, control electronics, and operation of both ACR I and ACR II has been discussed in many other publications [1, 4–11]. A comparison of the specifications of these two ACR types is presented in Carter et al. (2005) [1]. Here we will only point out a few of the salient features of the two ACR types. We pay particular attention to features relevant to the present work. A diagram of the ACR is depicted in Fig. 1.

Both ACR types have a conical shaped receiver cavity constructed of very high purity electrodeposited copper. The receiver cavity is coated with high emissivity specularly reflective black paint, Aeroglaze Z302^1^. At λ=632.8 nm, the cavity absorptance of ACR I has been measured to be 0.9987 [1]. The cavity absorptance was measured to be 0.99986 [12] for an ACR with a similar design to that of ACR II, and is close to the estimated value of 0.9993 [1]. In both ACR types there is a receiver cavity that has a thermal mass that is connected to a heatsink via a thermal link that has a thermal impedance greater than that of the copper receiver cavity. To improve the time response, the thermal mass of the ACR II receiver cone was reduced to less than 20 % of that of ACR I. To improve sensitivity, the stainless steel heatlink on ACR I was replaced with polyimide, which has a thermal resistance of about 10 times greater than that of stainless steel at 4 K [13]. The noise floor of ACR II is lower than ACR I, the responsivity is higher, and the time constant is shorter at low power levels. In this study, a defining aperture of 2 cm in diameter was placed in front of the ACRs. The ACR Is have two germanium resistive thermometers (GRT) symmetrically placed on the outside of the receiver cone, near the apex. Two resistive heating elements are also attached to the outside of the cone. One is placed near the apex, the other near the base. Only one heating element and one GRT are used to make a power measurement. The other set is a spare and can be used to address electrical non-equivalence, discussed later in this paper. Of the ACR IIs, only the newer one, dubbed ACR IIb, has two GRTs and two heaters. These are mounted in pairs on the outside of the receiver cone. One pair (GRT #1, heater #1) is mounted near the apex of the cone, whereas the other pair (GRT #2, heater #2) is mounted near the base of the cone, closer to the polyimide thermal link. The older ACR II, the ACR IIa, has only one GRT and one heater located close to the thermal link. Electrical non-equivalence cannot be investigated for ACR IIa.

The heatsink is constructed of oxygen free high conductivity copper. It is much more massive than the receiver cone, and is directly bolted to a copper block that is attached to a liquid He reservoir maintained near 2 K. To reduce thermal fluctuations, the heatsink is operated at a constant temperature slightly higher than that of the He reservoir. The temperature is controlled by a resistive heating element and is monitored by a GRT. Both are directly bonded to the heatsink by low-temperature epoxy.

The same control electronics are used for both ACR types. The receiver and heatsink heaters are controlled by the electronics to maintain a constant temperature as determined by the resistance of the two respective GRTs. A sensitive AC bridge is used to monitor the GRT resistance. The electric power dissipated in the receiver heater is precisely determined by the control electronics. The optical power from a laser beam, or the radiation power from a blackbody source, is determined from the difference between the average receiver heater power measured with radiation striking the receiver cone and that measured with the radiation source blocked. This value is then adjusted by the cavity absorptance to obtain the final radiative power striking the ACR.

### 2.2. SCC

All of the ACR-to-trap calibrations were performed in the LBIR Spectral Calibration Chamber (SCC), described in Carter et al. 2003 [11]. The main volume of the test chamber is a stainless steel cylinder 50 cm in diameter and 125 cm in length. A stainless steel cylindrical inner cryo-shroud surrounds the test volume. During the ACR-to-trap detector intercomparison, the cryo-shroud is cooled down to 15 K by a closed loop He refrigeration system. A small hole, 2 cm in diameter, in the end of cryo-shroud opposite to the ACR entrance aperture and 0.8 m from the ACR allows the laser beam to enter the test volume after passing through a Brewster window. The Brewster window is located about 1 m from the hole in the cryo-shroud. The chamber volume between the cryo-shroud and Brewster window is not cooled and therefore operates at ambient temperatures. We estimate that approximately 30 µW of ambient background radiation is seen by the ACR through the hole in the cryo-shroud. A door in the side of the SCC and a removable panel in the cryo-shroud allow easy access for placing and removing the trap detector during the Brewster window transmittance measurement, discussed below.

Before the cryo-shroud is cooled, the chamber is initially evacuated by a combination of turbo pumps and cryo-pumps. These pumps are not necessary once the cryo-shroud is cool. The vacuum pumps are generally not run during a data cycle to reduce noise. The ultimate pressure achieved in the chamber outside of the cryo-shroud is typically in the low 10^−5^ Pa range.

### 2.3 Trap Detector and Calibration by the Primary National Standard

A QED-150 trap detector was used as the transfer standard in this study. This is a double pass, three Si photodiode reflectance trap detector. The optical path within the detector includes five reflections so that the total reflectance at λ=632.8 nm should be less than 0.4 % [14]. In any case, this was accounted for in the calibrated external responsivity. To determine an absolute external responsivity, the optical power from an intensity stabilized He-Ne laser was first measured using the HACR [14,15] and then again, at a later date, by the POWR. For both instruments, the laser beam enters through a quartz window set at the Brewster angle. The transmittance of this window was determined in a separate experiment and ultimately limits the accuracy of the trap detector when calibrated against the HACR. Unlike HACR, POWR does not require the quartz window to be moved and fully removed from the apparatus for a transmittance measurement; the window transmittance measurement did not limit the uncertainty of the POWR measurements reported here, as it did the HACR. The trap detector was then placed in the path of the laser beam. Since the absolute optical power of the laser beam is known from the primary standard, the current produced from the trap detector determines the external responsivity. The trap used in this study was paired with a transimpedance amplifier set at a gain of 10^4^ V/A. A precise measurement of the voltage output of the transimpedance amplifier determines the output current of the trap detector. The external responsivity of the trap was thus determined to be 0.50745 A/W at the beginning of this effort, and 0.50746 A/W at the end. The later value is used in the final analysis presented in this paper. The uncertainty associated with the calibrated responsivity is 0.02 % (*k* =1). Additional details of the operation of the HACR instrument and of trap detector calibrations can be found in references [2,14,15].

### 2.4 Optical Setup

**Fig. 2:**
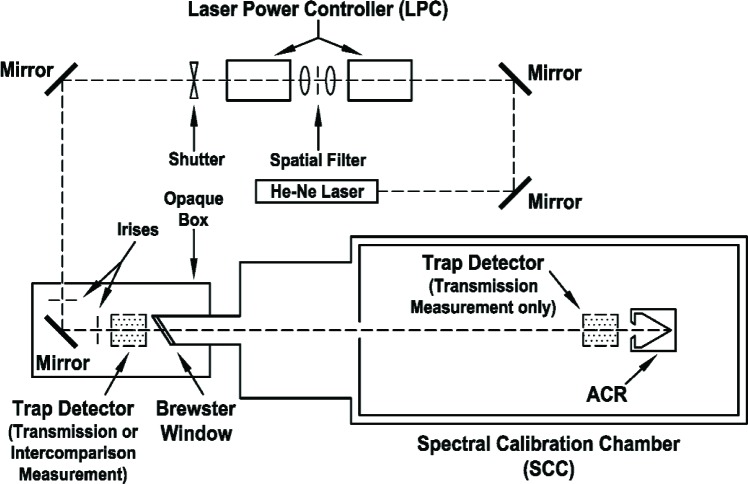
Schematic diagram of the optical arrangement used for the intercomparison between an ACR maintained by the LBIR facility and a trap detector. The same optical arrangement was also used to measure the effective window transmittance. The trap detector was not placed inside the SCC during an intercomparison measurement.

The experimental arrangement used in this study is shown in Fig. 2. The optical arrangement evolved somewhat over the course of this study. Improvements were made that decreased the uncertainties in repeatability and noise. The arrangement shown was used for both ACR I intercomparisons and was similar to that used for both ACR II intercomparisons. Differences between the two only affected the total random uncertainty and ease of operation, but not the accuracy of the experiment.

The light source for the intercomparison was a polarized He-Ne laser with a maximum power output of 1.5 mW. The polarization extinction ratio of the He-Ne laser was checked and is less than 1 × 10^5^. No polarizer external to the He-Ne laser was used in the optical setup. Two folding mirrors were used to direct the output of the laser into the first element of a laser power controller (LPC). The LPC consisted of two elements: the first element contained an electro-optic modulator which reduces and stabilizes the laser power; the second contained a beam splitter and a photodiode used to monitor the laser power and provide feedback to the electro-optic modulator. A spatial filter was placed between the two optical elements of the LPC. Placing the spatial filter after the electro-optic modulator, but before the feedback photodiode, insured that the spatial filter removed distortions and scatter caused by the modulator while still allowing the LPC servo to control on filtered laser light. The spatial filter consisted of a 10X microscope objective that focused the laser light through a 50 µm diameter pinhole aperture after which the light was re-collimated by a 5X microscope objective. The power of the microscope objectives were chosen to expand the beam profile in order to reduce the effects of non-uniformities in the silicon trap detector and make the beam diameter similar to that which was used to calibrate the trap detector against the primary standard. No other transmissive optical elements were used in the optical train. The diameter of the laser beam at the silicon trap detector and the ACR was typically about 2.5 mm.

After the beam exited the last element of the LPC, two folding mirrors directed the beam through a 57° quartz Brewster window and into the vacuum chamber. The ACR was located more than 2.25 m from the last folding mirror. Irises were placed on each side of the last folding mirror to reduce scattered and stray light from entering the ACR or trap detector. When the trap detector was mounted outside of the vacuum chamber, it was placed between the last folding mirror and Brewster window. In this position, the trap, along with the last folding mirror and Brewster window, was surrounded by an opaque box which prevented most of the ambient light from reaching the trap. Background signals were measured by blocking the laser light immediately after the LPC.

The trap detector was directly mounted to a 2-axis tilt stage which was, in turn, mounted on a kinematic base. The trap detector tilt was adjusted such that the retro-reflected beam diverged from the incoming beam by approximately 2 mrad. By adjusting the tilt in this way, the retro-reflected laser beam did not pass back through the LPC apparatus, but the trap detector was still sufficiently aligned for accurate power measurement. The bottom plate of the kinematic base was mounted on a two-axis translation stage and was permanently positioned between the last folding mirror and Brewster window. A second kinematic bottom plate with a two-axis translation stage was permanently mounted in front of the ACR and was used during the window transmittance measurements. This mounting system was used so that the trap detector could be reproducibly removed and replaced. The technique used for the alignment was similar to that described by Houston and Livigni (2001) [15]. The trap detector was aligned vertically and horizontally by centering it between the points where the measured optical power dropped to 80 % of the peak. Only a minor adjustment of the tilt was required when the trap detector was moved between the two positions.

Several improvements in the optical setup led to better repeatability and hence lower uncertainty than in previous intercomparison attempts. First, the use of non-reflective optical elements such as polarizers and lenses, particularly after the spatial filter, was minimized or eliminated to reduce scattered light. Second, enclosing the trap, last folding mirror, and the Brewster window in an opaque box greatly reduced scattered light from ambient sources. This also enabled the experiment to be performed with the laboratory lights on, which not only was convenient but eliminated laboratory temperature changes which occur from cycling the laboratory lights on and off. Laboratory temperature changes did have a small but noticeable effect on the laser power measurement by the trap detector. Third, the use of kinematic mounts assured the reproducible placement of the trap in its two locations, which made the evaluation of the uncertainty due to trap alignment reliable.

## 3. Measurement Method

Since the ACR must be operated in a cryogenic environment whereas the Si trap detector is operated in the ambient laboratory environment, two steps are involved in performing an intercomparison between a calibrated Si trap detector and the ACR.

First, the fraction of laser light reaching the ACR from outside the chamber must be determined. There is some loss of laser power due to the transmittance of the Brewster window. In addition, there are other losses or gains in the detected laser power which occur between the location of the trap in front of the Brewster window and the entrance of the ACR aperture, such as light from the laser that is scattered in to or out of the field of view of the trap detector. All such effects were collectively taken into account by measuring a transfer function or an effective window transmittance. The effective window transmittance was determined by taking the ratio between optical power measurements made with the trap detector placed just in front of the ACR entrance aperture to those made with the trap detector placed in front of the Brewster window. This provides the necessary relationship between the laser power measured directly in front of the Brewster window to that in front of the ACR entrance aperture when the ACR is under test conditions.

Second, the absolute optical power determined with the calibrated Si trap detector is compared to the optical power determined from the ACR. This is done by comparing the laser power directly in front of the Brewster window, measured with the trap, to that measured inside the vacuum chamber with the ACR. The comparison equation is:

$$R_{\text{trap}/\text{ACR}}=T\cdot \left(\frac{P_{\text{trap}}}{P_{\text{ACR}}}\right)$$ (1)

where *T* is the effective window transmittance, and *P*_trap_ and *P*_ACR_ are the radiometric powers measured by the trap detector and ACR, respectively. Since trap and ACR measurements are always measured in pairs, it is convenient to define the parenthesis in (1) as 1/*R*_0,ACR/trap_, so that

$$R_{\text{trap}/\text{ACR}}=T/R_{0,\text{ACR}/\text{trap}}$$ (2)

*R*_trap/ACR_ then consists of two separate measurements: an effective window transmittance measurement and a ratio of the optical power measured with the ACR to the optical power measured with the trap detector. Details of the transmittance measurement are in the following section.

The ratio *R*_0,ACR/trap_ was measured for several different laser powers for each ACR considered in this study. Trap detector radiometric power measurements were made outside of the vacuum chamber in front of the Brewster window, as described in the previous section. The trap detector was on a kinematic mount and could quickly be removed from or inserted into the laser beam path in a repeatable fashion. Background signals in the trap detector or ACR were measured by blocking the laser beam just after the second element of the LPC. Radiometric power measurements with the ACR have been described in several publications [4,5,8]. The radiometric power measured by the ACR is determined by first recording data with the laser blocked, unblocking the laser, and then blocking the laser again. The background and laser-on data are separately fit to a straight line in order to remove drifts. The difference between the two power levels is the radiometric power measured in the ACR. The statistical error of the measurement is calculated from the fit.

At any given ACR receiver operating power, the ratio *R*_0,ACR/trap_ was typically measured 3–5 five times in varying order, similar to what was done for the effective window transmittance measurements. The ratio was measured at several different ACR operating powers for every ACR in this study. Measurements were repeated several times at one or more ACR operating powers so that long term repeatability could be checked. This was done at different times and days. For any given ACR, the intercomparison ratio, *R*_0,ACR/trap_, data were taken over 2–3 days.

Reproducibility was evaluated by taking the standard deviation for ratio measurements made at the same receiver operating power and laser power, but on different days or times. This uncertainty includes electrical noise and other random errors. In addition, the trap was re-aligned several times at a given ACR operating power and laser power to determine the uncertainty due to the trap detector alignment. These uncertainties, the uncertainty in the trap calibration, and the uncertainty in the window transmittance measurement were all added in quadrature to yield the total combined uncertainty in the intercomparison. Except for the uncertainty associated with the calibrated trap detector responsivity, all of the combined uncertainties are derived by statistical methods (Type A). The uncertainty associated with the trap detector responsivity was taken from an internal calibration report and is considered to be a Type B uncertainty [3,16]. Throughout this paper, uncertainties are provided as *k* =1.

Instability or drift in measured laser power can contribute to the measurement uncertainty. Measurements always consisted of a ratio of the radiometric power measured in the chamber either by the trap detector (during a Brewster window transmittance measurement) or by the ACR (during an intercomparison), to the power measured outside of the chamber by the trap detector. A single power ratio measurement typically took less than 10 minutes. The drift in this time period should have been small; nevertheless, the ratio was always measured several times in varying order. Therefore, the uncertainty calculated from the measurement repeatability takes drift into account. The LPC manufacturer’s specification on long term laser power control stability is 0.05 %. Our own observations confirm that the measured laser power varies by roughly 0.05 % over many hours of operation.

## 4. Window Transmittance Measurements

The effective window transmittance was determined by taking the ratio of the laser power measured outside of the vacuum chamber, immediately before the Brewster window, to that measured inside of the chamber, immediately in front of the ACR. The trap detector was used for this measurement. Kinematic mounts were used at both trap positions to ensure the reproducibility of the trap detector position. An effective transmittance measurement began with the alignment, described above, of the trap detector both inside and outside the chamber. Optical power measurements were then made both inside and outside the chamber. A single power measurement consisted of 10 to 25 voltage readings that were subsequently averaged together. Each reading was separated in time by five time constants, where the time constant is equal to the integration time of the voltmeter. An effective transmittance ratio data point consisted of a power measurement made inside the chamber divided by a power measurement made outside the chamber. Without changing the alignment, five transmittance data points were collected, varying the power measurement order (i.e. outside then inside, inside then outside, etc.). These five measurements were subsequently averaged together and were considered to be a single measurement of the effective window transmittance. The trap detector was then re-aligned, and the effective window transmittance measurement was again measured. This procedure was performed 2 to 3 times before and after each ACR inter-comparison. Thus, a total of 5 to 6 effective window transmittance runs were made for each ACR intercomparison performed. The effective window transmittance used in the data analysis of an ACR intercomparison was the average of the transmittance runs. The Type A (statistical) uncertainty, determined by standard error analysis and propagation techniques in this effective window transmittance measurement procedure, represents alignment uncertainty, electric noise, laser power drift, electronic drift effects, and other possible random errors. The calibrated responsivity of the trap is not relevant to the window transmittance measurement, and therefore makes no contribution to the uncertainty budget of the effective window transmittance determination.

The results of the effective window transmittance measurements are presented in Table 1 for the four ACRs in this study. Considering that the total time of this work was several months, the transmittance measurements show a surprising degree of consistency. Note that the window was typically not removed between the different ACR intercomparisons.

## 5. Intercomparison Data and Discussion

Although the intercomparison was investigated over a range of receiver powers, typically from 1 µW to 150 µW, no significant dependence on receiver power was observed in the final ratio *R*_trap/ACR_. The intercomparison data, *R*0,ACR/trap, for both ACR I and ACR II are summarized in Table 2. The results in Table 2 are corrected for cavity absorptance, but do not include the effective window transmittance. Table 2 also contains the uncertainty budget for the intercomparison ratio. Since the uncertainty associated with the cavity absorptance is much less than 0.01 %, it is not included. The ACR I intercomparison data shown in Table 2 are only the results of data collected at a laser power of 32 µW. For both ACR I intercomparisons, 4–5 data points at this power were taken at different times and days. Window transmittance data was also taken with the laser power set to 32 µW, for these two ACRs.

For both ACR IIs, the ratio *R*_0,ACR/trap_ was taken as an average of measurements made over several ACR operating powers and laser powers between 1 µW and 150 µW. No relevant dependence on laser power or receiver power could be seen in *R*_0,ACR/trap_ for either ACR II over this power range. The results are tabulated in Table 2. Except for the ACR IIa, the results in Table 2 are for the GRT and heater mounted nearest the apex of the cone in the ACR cavity. The laser strikes the interior of the receiver cone near the apex. The ACR IIa only has one working GRT, which is located near the cone base. All GRTs and heaters are mounted on the exterior of the receiver cone.

Final results for the intercomparison are tabulated in Table 3. These are derived by combining the *R*_0,ACR/trap_ in Table 2 with the effective transmittance measurements in Table 1 (see Eq. (2)). The combined uncertainty in Table 2 is combined in quadrature to the uncertainty in Table 1 to produce the total combined uncertainty in Table 3.

Since all of the ratios reported in Table 3 are less than unity, it is clear that there is some small systematic bias in the data. There are several possible sources of uncertainty or systematic bias that are not accounted for in Table 3. The ACR power measurements rely upon an accurate calibration of the electronics which are used to control and determine the electric power to the ACR heaters. The calibration of the electronics was checked, and it was found that the heater power determined by the control electronics was within +0.02 % of the actual heater power. Any discrepancy would cause *R*_trap/ACR_ to become smaller than the values reported in Table 3. An electrical non-equivalence, discussed in the following paragraph, can also cause a systematic uncertainty, but this was found to be small for all but the ACR IIa. The cavity absorptance, which affects the measured ACR power, may also systematically affect the determined ACR power and thus ratio *R*_trap/ACR_. The value for the absorptance that was used in the analysis represents the average absorptance of the ACR cavity. There may be small non-uniformities in the cavity absorptance. The diameter of the He-Ne laser beam used in this study is small compared to the ACR cavity, and so the absorptance seen by the laser beam may not be identical to the average cavity absorptance. It is also possible that a systematic bias can result from the spatial non-uniformity of the trap detector. Despite the efforts to follow the trap alignment procedure that was used for the responsivity calibration, there still may be subjective influence in the trap alignment procedure. Also, the spatial uniformity of the trap detector can be greatly affected by the cleanliness of the Si surfaces of the detecting elements in the trap detector. A small particle of dust on one of the inner Si detectors that is not visible from the outside of the detector could change the efficiency of the detector if it is centered on the trap detector; or it could change the alignment of the trap detector if it happens to be near one of the 80 % power points that are used to align the detector. For present analysis, it was assumed that effect of the alignment differences between the primary national standard measurements and ACR measurements were negligible. It is also assumed that the surface cleanliness of the Si photodiode did not change over the course of the intercomparison activity. These assumptions are supported by the close agreement of the trap detector responsivity measured before and after the intercomparison activity by the HACR and POWR, respectively. Finally, it is possible that a systematic bias in the effective window transmission measurements resulted in a bias in the final intercomparison ratio. For example, the ACRs have a larger angle of acceptance than does the trap detector. It is therefore possible that the ACRs collect more scattered light during the power measurement than does the trap detector during the effective window transmission measurement. This would result in effective window transmission measurement that is too small. Additionally, the Brewster window will flex slightly when the chamber is placed under vacuum. It is possible that this will slightly change the effective window transmission. It is exceedingly difficult to determine the relevance of these effects.

There is a measurable non-equivalence between the change in optical power entering the ACR and the corresponding change in electrical power required to maintain a constant receiver cavity temperature. Electrical power lost to heat in the leads is reduced to negligible levels by using superconducting Nb wire for the receiver heater leads. The difference in power distribution (electric heating versus optical heating) affects the temperature distribution in the receiver cavity and may cause an error in the power measurement. With the exception of the ACR IIa, all of the ACRs in this study were equipped with two GRTs and two heaters. One GRT and heater were mounted near the apex of the cavity (GRT #1 and heater #1) furthest from the thermal link to the heat sink, whereas the other GRT and heater were mounted near the base of the cone (GRT #2 and heater # 2) adjacent to the thermal link to the heat sink. Thermal non-equivalence is checked by comparing radiant power measurements made for all heater and GRT combinations relative to the radiant power measured by the trap detector. Since ACR IIa has only one working GRT and heater on the receiver cavity, this check could not be made for the ACR IIa.

For ACR Ia the thermal non-equivalence was checked at 10 and 40 µW. Power measurements made using all possible heater/GRT combinations. The non-equivalence was determined by taking the maximum difference between all of these power measurements. For ACR Ib, this was checked at 10, 40, and 80 µW. The non-equivalence was observed to be less than 0.02 %, which was within the statistical error of the measurement. For ACR IIb, the results were somewhat different and are summarized in Table 4.

As can be seen in Table 4, for ACR IIb the results of GRT #1 and heater #2 are significantly different than the three other possible configurations. Considering only GRT #2, the non-equivalence between the two heaters is 0.022 %. Similarly, the non-equivalence for GRT #1 is 0.64 %. The ACR II was expected to have a higher non-equivalence than ACR I: the thinner walls of the receiver cavity in ACR II results in larger thermal gradients in the receiver walls. During the optical power measurements, the laser beam strikes near the apex of the receiver cone near GRT #1 and heater #1. When heater #1 is used for the electrical power substitution, the thermal gradient created by heater #1 should be similar to that created by the laser. Nevertheless, the laser does not strike in the exact position of heater #1 nor is the heat load distributed in the same way. As can be seen from Table 4, there was a measurable difference of 0.092 % for the intercomparison values for heater #1 between GRT #1 and GRT #2. This demonstrates that the thermal gradient in the cavity walls caused by the use of heater #1 is not the same as that caused by the laser heating, resulting in a power measurement error. GRT #2 is located in a position that is less sensitive to temperature gradient differences because it is close to the thermal link and is further from both where the laser strikes the cavity and the position of heater #1 than is GRT #1. At that position the temperature of the receiver cavity wall is predominately determined by the power through the thermal link multiplied by its thermal impedance and added to the temperature of the heat sink. On the other hand, GRT #1 is more sensitive to temperature gradient differences in the cavity wall than is GRT #2 because it is not close to the thermal link, and as a result the temperature determined by GRT #1 is more influenced by thermal gradients in the receiver cavity walls than is GRT #2. The result of this thermal non-equivalence is that the optical power determined using the combination of GRT #1 and heater #2 is larger than the optical power actually applied. Although a correction factor is typically applied to calibrations performed by the ACR, GRT #2 is preferable to GRT #1 for the reasons cited above.

## 6. Summary

The four ACRs maintained by the LBIR facility have been compared to a NIST primary standard. The results of this intercomparison are in accord with the uncertainty of the intercomparison technique. From Table 3, we can conclude that the absolute power accuracy of the ACRs compares to that of the primary standard to within about 0.1 %. The thermal non-equivalence for three of the four ACRs were measured and it was found that the ACR I type radiometers have a thermal non-equivalence that was about six times smaller than the ACR II type radiometers.

## Figures and Tables

**Table 1:** Effective window transmittance determined for the intercomparison measurements

ACR intercomparison	Effective window transmittance *T*	Type A uncertainty (*k* =1)
ACR Ia	0.998561	0.000065
ACR Ib	0.998535	0.000145
ACR IIa	0.998325	0.000416
ACR IIb	0.997358	0.000015

**Table 2:** Ratio of radiometric power measured in an ACR to that measured in a calibrated trap detector (*R*_0,ACR/trap_), and the associated uncertainties. This ratio is not corrected for effective window transmittance. The uncertainties associated with the repeatability and alignment are Type A, whereas the trap responsivity uncertainty is Type B. All reported uncertainties are for *k* =1.

ACR	*R*_0,ACR/trap_	Repeatability	Alignment	Trap responsivity	Combined
ACR Ia	0.999222	0.0059 %	0.0069 %	0.021 %	0.023 %
ACR Ib	0.999405	0.0068 %	0.0145 %	0.021 %	0.026 %
ACR IIa	0.998730	0.0057 %	0.0250 %	0.021 %	0.033 %
ACR IIb	0.998592	0.0039 %	0.0200 %	0.021 %	0.029 %

**Table 3:** Final Intercomparison Ratio. Except for ACR II(a), which only has one GRT near the cone base of the cavity, all results are for GRT #1 and heater #1, mounted near the apex of the cone.

ACR	*R*_trap/ACR_	Total combined uncertainty (*k* =1)
ACR I(a)	0.999339	.024 %
ACR I(b)	0.999130	.030 %
ACR II(a)	0.999595	.053 %
ACR II(b)	0.998765	.029 %

**Table 4:** Non-equivalence for ACR IIb.

GRT	Heater	*R*_0,ACR/trap_	Repeatability
1	1	0.998592	0.0039 %
1	2	1.005046	0.0108 %
2	1	0.997677	0.0134 %
2	2	0.997901	0.0135 %

## Footnotes

1 Certain commercial equipment, instruments, or materials are identified in this paper to foster understanding. Such identification does not imply recommendation or endorsement by the National Institute of Standards and Technology, nor does it imply that the materials or equipment identified are necessarily the best available for the purpose.
